# Age-Related Changes in Mobility Evaluated by the Timed Up and Go Test Instrumented through a Single Sensor

**DOI:** 10.3390/s20030719

**Published:** 2020-01-28

**Authors:** Giulia R.A. Mangano, Maria S. Valle, Antonino Casabona, Alessandro Vagnini, Matteo Cioni

**Affiliations:** 1 Laboratory of Neuro-Biomechanics, Department of Biomedical and Biotechnological Sciences, School of Medicine, University of Catania, 95123 Catania, Italycasabona@unict.it (A.C.); mcioni@unict.it (M.C.); 2 Residency Program of Physical Medicine and Rehabilitation, Department of Biomedical and Biotechnological Sciences, University of Catania, 95123 Catania, Italy; 3 BTS Bioengineering, 20024 Garbagnate Milanese, Italy; alessandro.vagnini@btsbioengineering.com

**Keywords:** instrumented timed up and go test, wearable sensor, mobility, turning, aging

## Abstract

Mobility across people with a large range of age was evaluated, for the first time, by using an instrumented timed up and go test (iTUG) based on signals acquired by a single wearable inertial sensor. Eighty healthy participants, from childhood to old age, performed the test, covering walking distances of 3 m and 7 m. Total time, temporal, and velocity parameters of linear and turning subcomponents of the test were quantified. While children, adults, and senior adults exhibited similar values for all the parameters, older adults showed increases in duration and reductions in velocity during the turning phases when compared with the other groups. an increase in velocity was observed during mid turning when the test was performed along the longer distance. Similarity across children, adults, and senior adults indicates that healthy individuals develop the abilities performed in the iTUG early, while the slowing down shown during the turning phases by the older adults may reflect the need to implement adaptive adjustments to face changes of direction. These results emphasize the idea that reducing equipment to a single sensor provides an appropriate quantification when the iTUG is used to investigate a broader age range or different levels of complexity.

## 1. Introduction

The increase in life expectancy resulted in an inevitable aging of populations; thus, there is growing interest in the study of mobility associated with healthy aging. Human mobility changes throughout life via a progressive development of a repertoire of abilities acquired in childhood, until there is a gradual reduction and deterioration of physical capability in the elderly.

Several tests were designed to evaluate age-related gross motor functions, such as walking [1,2,3], balance [1,3], standing up from a sitting position [4] and turning around an obstacle [5]. A limitation of these tests is that each motor skill is studied as a single action, while, in everyday life, these actions are combined to generate a natural sequence of movements.

The timed up and go test (TUG) is a simple test that overcomes these limitations, embedding several actions into a natural sequence. In fact, individuals have to stand up from a chair, walk forward, turn around an obstacle, walk back to the chair, and sit down. In the most basic use of the TUG test, the distance from the starting position to the turning point is 3 m, and the level of mobility is quantified by taking the time to travel the entire path (total time). The variety of the subcomponent tasks integrated in a single context makes the TUG test one of the most common tools for the assessment of a general level of physical mobility in the elderly and in a number of specific pathologies, such as Parkinson’s disease [6,7], stroke [8], multiple sclerosis [9], muscular dystrophy [10], and cerebral palsy [11,12].

The application of this test in healthy people is mainly focused on the elderly without gait disturbances or with increased risk of falling [13,14,15,16]. Only in some papers was the TUG test applied to typically developing children [17,18,19] and young adults [14,20].

The prevalent use of the TUG test in the elderly reduced the possibility of this test drawing the entire picture of mobility from childhood to old age. This issue was raised by Lara et al. [21] in a review paper where a number of tests assessing mobility were compared. These authors noted that the limited age range precluded the TUG test from being considered a comprehensive tool for the evaluation of physical capability.

An important factor contributing to the above limitation is the low sensitivity of the traditional TUG test in distinguishing between populations [22]. In fact, as the total time can be considered a good outcome to represent general mobility, differences or similarities in the total time across people categories can be interpreted only by identifying the contribution of each subcomponent. For example, possible difficulties of older adults to face turning with respect to younger adults would only emerge if, in addition to the total time, the single components were measured.

Technological advances over the last few decades met the need to improve the parameterization of the TUG test, developing an instrumented version of this test (iTUG). Motion analysis systems, such as optoelectronic systems [23] and wearable inertial sensors [16,22,24], provide accurate kinematic measurements, detecting the velocity and the acceleration of the whole body or single segments. 

The reduced dimension and the wearability of inertial sensors recently made the use of these technologies easier and less expensive. Two or three sensors are often simultaneously mounted on the body to record movements [25]. Up to now, there were few reports concerning the study of subcomponents during the iTUG test achieved by a single wearable sensor attached to the lower back [20,26]. Similarly, modern smartphones can be used as single sensors, stimulating even more the diffusion of the instrumented version of the TUG test [27,28].

Thanks to this advancement, iTUG provides an accurate quantification of the subcomponents and facilitates the interpretation of the functional differences when various ages or conditions are compared. 

With this in mind, in the current paper, we report our use of a single sensor to study a large range of ages and two different walking distances, trying to show the possibility of the iTUG to be a comprehensive test for assessing physical mobility. In particular, we decided to involve a group of children (7–12 years old), young adults (31–40 years old), senior adults (60–69 years old), and elderly people (71–85 years old), measuring the total time and the duration and velocity of each iTUG subcomponent.

In addition, we compared the results from the traditional 3 m walking distance with those obtained from a path of 7 m, with the aim of revealing possible effects of a longer linear walk on the results of the tests.

## 2. Materials and Methods

### 2.1. Subjects

This was a transverse study conducted on 80 healthy volunteers, divided into groups of different age: 19 children (7–12 years old; nine males), 20 young adults (31–40 years old; 10 males), 20 senior adults (60–69 years old; 10 males), and 21 elderly people (70–85 years old; 10 males). The complete anthropometric data are reported in the Table 1 as average ± standard deviation for each group. The inclusion criteria for participating in the study required a normal or corrected to normal vision and being in good health. The exclusion criteria were as follows: current practice of competitive/professional sports, neurological or orthopedic disturbances, cognitive disorders or any other disorder that could affect balance or mobility and a height under or over the 25th or 75th percentile. Before the test, a clinical examination was performed to ascertain the baseline functional status, evaluating the range of motion of the spine and lower limbs and measuring muscle strength using the scale of the Medical Research Council [29].

All participants received standardized instructions and explanations about the experimental set-up so that equipment and rules were the same for everyone.

The adults and the legal guardians of individuals younger than 18 years old signed an informed consent form before starting the study. The research project was approved by the local ethics committee of Catania University Hospital “Policlinico-Vittorio Emanuele” (n° 209/2019/PO), and all participants or their legal guardians of individuals younger than 18 years old provided written consent to participate. All procedures were performed according to the Declaration of Helsinki.

### 2.2. Experimental Procedure

**Figure 1:**
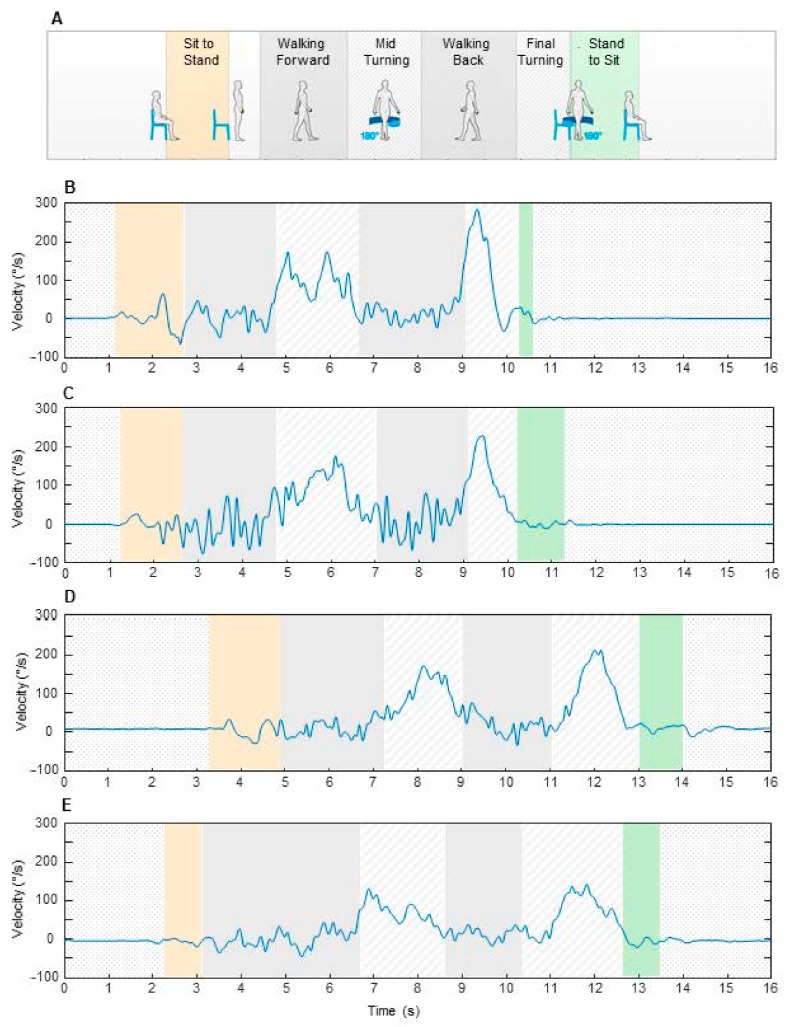
Subcomponents of instrumented timed up and go (iTUG) test measured in the experiments (**A**). The plots represent examples of angular velocity profiles and components duration observed in a child (**B**), a young adult (**C**), a senior adult (**D**), and an elderly person (**E**). The data are relative to the test performed along 3 m.

All participants performed the iTUG test with bare feet and with comfortable clothes in order to ensure adequate mobility. They executed the following sequence of simple tasks: stand up from a chair 46 cm high, walk along a straight path covered in a rubber runner, turn 180° around a pin 18 cm high, walk back to the chair, turn, and sit down again (Figure 1A). The individuals performed the test covering both a typical distance of 3 m from the chair and an extended distance of 7 m.

For the group of children, we used an adjustable chair to guarantee that each child started from a standard position with approximately 90° hip, knee, and ankle flexion.

All participants were asked to walk at their own normal pace. The test was repeated three times for each distance, and the median value was used for the subsequent analyses.

### 2.3. Data Collection and Processing

We used a commercial wearable inertial sensor (G-Sensor, BTS Bioengineering, Italy) applied over the skin of the second lumbar vertebra. The sensor was composed of a triaxial accelerometer 16 bit/axes (sensor range, ±2 g), a triaxial magnetometer 13 bit (±1200 μT), and a triaxial gyroscope 16 bit/axes (sensor range, ±2000°/s). The signals were sampled with a frequency of 100 Hz. The weight of the sensor was 37 g, with dimensions of 70 × 40 × 18 mm. 

Acceleration data were transmitted via Bluetooth to a laptop computer for acquisition and processing using a dedicated software package (BTS® G- Studio, BTS Bioengineering, Italy). Sensor signals were used to subdivide the TUG test into six sequential subcomponents as follows (Figure 1): sit to stand, walking forward, mid turning, walking back, final turning, and stand to sit. The subcomponents were identified using the criteria described by Negrini et al. [30]. Then, the following eleven movement parameters were considered: total time of the iTUG test, duration of the single subcomponents, average and peak mid turning velocity, and average and peak final turning velocity. 

Considering that the variability in body dimensions is particularly marked in children with respect to the other groups, the values of the original measurements were normalized following the suggestions of Wahid et al. [31] and Pinzone et al. [32]. These authors showed that a non-dimensional normalization, based on the multiple-regression method, is the most appropriate in normalizing gait data. By using this approach, body weight and height were decorrelated from discrete iTUG measurements, gaining a maximization of the effects of age and distance. The process of transformation of the original un-normalized data to normalized parameters was performed as described below [31].

Firstly, from each original parameter (*P_o_*), a linear regression model was used to compute the best fit to determine the predicted parameter (*P_p_*), given weight (*W*) and height (*H*) as independent variables.

(1) $$P_{p}=\;\beta_{0}+\beta_{1}W+\;\beta_{2}H+\varepsilon ,$$

where *β_0-2_* are the regression coefficients, and *ε* is the independent residual error. 

Then, the level of correlation, expressed by the coefficient of correlation (*r*), with the level of statistical significance set at α < 0.05, was used as a reference to estimate the level of influence of weight and height in determining the *P_p_*.

Finally, in order to reduce this influence, the *P_p_* was decorrelated from weight and height by dividing *P_o_* by *P_p_*, according to the following equation:

(2) $$P_{n}=\;\frac{P_{o}}{P_{p}},$$

where *P_n_* represents the normalized parameter. 

The un-normalized and normalized data were collected in a specific database for the successive elaboration. 

### 2.4. Statistical Analysis

The statistical analysis was conducted on the normalized data. Parametric statistical analysis was adopted after preliminary tests for normality (Shapiro-Wilk test) and equality of sample variances (Levene’s test) were performed. Significant changes over the groups and between the distances were analyzed using a two-way analysis of variance (ANOVA) with group as the between-subjects factor and distance as the within-subjects factor. Post hoc analysis with Bonferroni correction was used for pairwise comparison between the groups.

The F-statistic was adjusted applying Greenhouse-Geisser correction, which produces a more conservative *p*-value. This procedure is typically applied to the repeated-measures ANOVA to correct the result with respect to a possible violation of the sphericity assumption. This assumption was checked by the Mauchly’s test.

The level of statistical significance was set at α < 0.05.

## 3. Results

Figure 1B–E show examples of angular velocity profiles measured around the vertical axis in four participants, with one from each group. The signal was distributed over six boxes, each representing single subcomponents, with the box width corresponding to the subcomponent duration. Although temporal and velocity parameters are expressed in un-normalized form, some changes showed in the plots have a quantitative relevance for the statistical analysis described in the next paragraphs. In particular, when comparing the elderly person with the other participants (Figure 1E), we found an increase in duration of the final turning and a reduced level of angular velocity during both mid and final turning. 

The statistical analysis was conducted on the data after normalization for weight and height (Equations (1) and (2)). The un-normalized data are reported in Table 2 for the temporal parameters and in Table 3 for the velocity parameters. At the bottom of the two tables, the values of correlation (*r*) and the level of significance (*P*) of the multiple regression model are reported, where each parameter was predicted by the weight and the height before and after data normalization. 

Temporal and velocity parameters showed middle/high levels of correlation, with significant *p*-values, except for walking forward over 7 m and walking back over 3 m. After performing the normalization procedure, all the values of correlation reduced to a very low level (including walking forward and walking back) with no significant *p*-values. However, walking forward and walking back showed a low level of correlation without normalization; to conform the analyses, we preferred to use the normalized data for all statistical computations reported in the subsequent sections.

### 3.1. Changes in Total Time and Linear Subcomponents of iTUG

**Figure 2:**
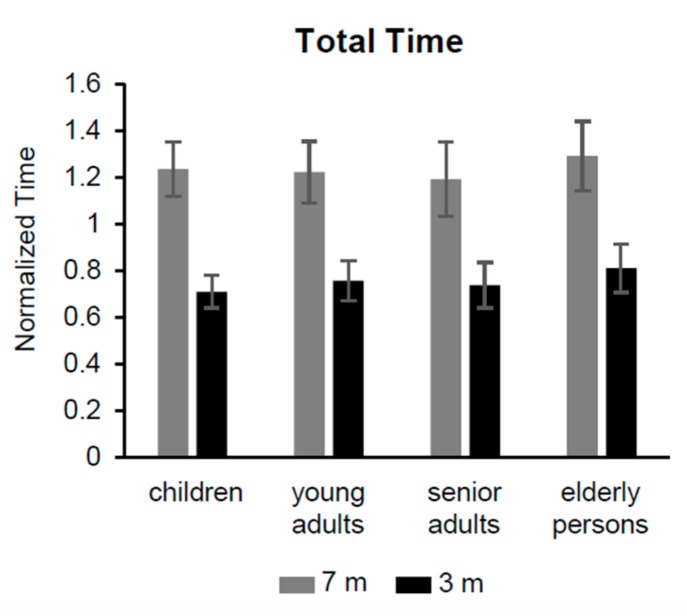
Total time of iTUG test. Non-dimensional normalized time of the four groups to complete the iTUG test over 7 and 3 m.

The iTUG total time from all groups showed differences with marginal significance (Table 4, row 1; Figure 2), with a trend where the elderly group showed a higher duration than the other groups.

An obvious significant different duration was found between the distances of 7 (1.24 ± 0.14 s) and 3 m (0.75 ± 0.10 s), and there was also a significant difference for the interaction between groups and distances. This interaction depends on the progressive reduction of the lag between the two distances, passing from children to senior adults; the values of the total time were progressively reduced across the distance of 7 m, while a progressive increase was observed for the distance of 3 m. The elderly group did not follow this pattern exhibiting a parallel increase of the total duration for both distances.

Considering the duration of the linear subcomponents of the iTUG test, the factor group showed no significant differences for the sit to stand duration (Table 4, row 2), as well as the time for walking forward (Table 4, row 3), for walking back (Table 4, row 4), and for stand to sit (Table 4, row 5).

The differences between the two distances were obviously significant for walking forward (7 m: 1.42 ± 0.22 s; 3 m: 0.59 ± 0.10 s; Table 4, row 3) and back (7 m: 1.46 ± 0.20 s; 3 m: 0.55 ± 0.14 s; Table 4, row 4), but the changes in duration of sit to stand (Table 4, row 2) and stand to sit (Table 4, row 5) were not significant for distance. 

No significant differences were detected for the interaction factor over all the linear subcomponents (Table 4, rows 2–5).

### 3.2. Changes in Duration and Velocity over the Turning Phases of iTUG

**Figure 3:**
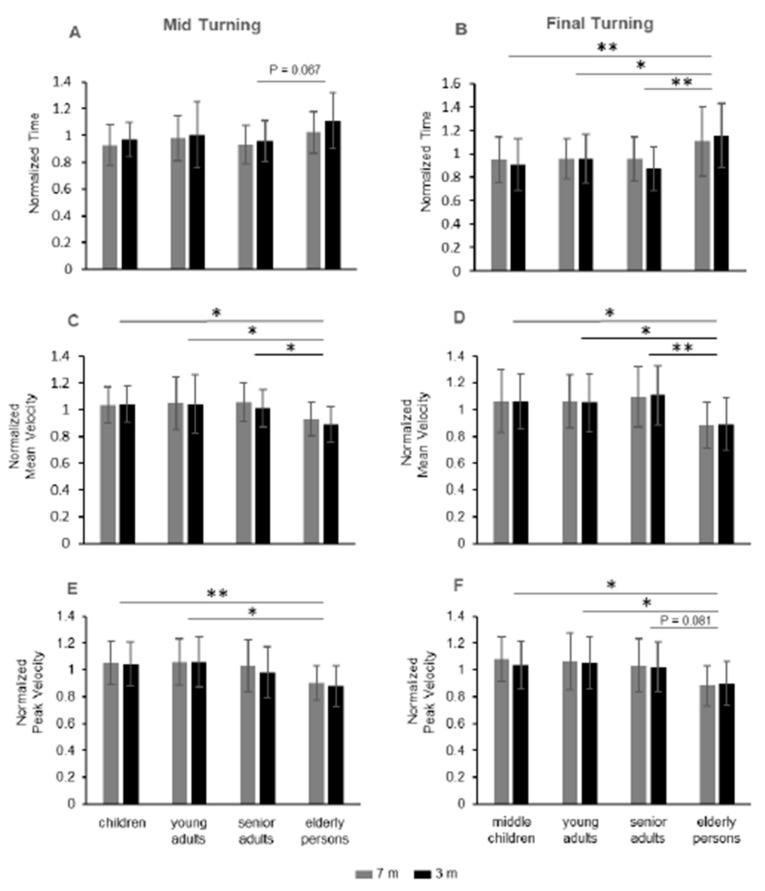
Duration and velocity in the turning phases of 7 and 3 m TUG. Non-dimensional normalized data of the four groups for the time in mid turning (**A**) and in final turning (**B**), for the mean velocity in mid turning (**C**) and final turning (**D**), and for the peak velocity in mid turning (**E**) and final turning (**F**). * *p* < 0.05; ** *p* < 0.01.

The factor group showed significant changes in duration during mid turning (Table 4, row 6; Figure 3A) and final turning (Table 4, row 7; Figure 3B). The mid turning duration was higher in the elderly group than senior adults, with marginal significance (*p* = 0.067; Figure 3A), while the duration of the final turning (Figure 3B) was significantly higher in the elderly group when compared with each of the other groups (elderly group vs. children: *p* = 0.007; elderly group vs. young adults: *p* = 0.029; elderly group vs. senior adults: *p* = 0.003). 

Thus, for the final turning, children, young adults and senior adults showed similar temporal values, while measurable changes were focused on the elderly group with more time than each of the other groups.

The differences in duration between the two distances were significant only for the mid turning duration (7 m: 0.96 ± 0.15 s; 3 m: 1.01 ± 0.19 s; Table 4, row 6).

No significant effects were detected for the interaction factor for both mid and final turning durations.

The velocity parameters showed significant changes over the groups during mid and final turning for both average velocity (Table 4, rows 8, 9; Figure 3C,D) and peak velocity (Table 4, rows 10, 11; Figure 3E,F).

Post hoc analysis revealed that the main contribution to the changes in velocity over the groups depended on the paired comparisons between the elderly group with respect to each of the other groups for mean velocity (Figure 3C,D), and between the elderly group compared with the children and the young adults for the peak velocity (Figure 3E,F). For the mid turning mean velocity (Figure 3C), the elderly group showed a lower value than senior adults (*p* = 0.048), young adults (*p* = 0.024), and children (*p* = 0.041). For the final turning mean velocity (Figure 3D), the elderly group showed a lower value than senior adults (*p* = 0.003), young adults (*p* = 0.03), and children (*p* = 0.027). 

For the mid turning peak velocity (Figure 3E), the elderly group showed no significant differences with respect to senior adults (*p* = 0.148), but their peak velocity was significantly lower than in young adults (*p* = 0.015) and in children (*p* = 0.008). For the final turning peak velocity (Figure 3F), the elderly group showed marginally significant differences with respect to the senior adults (*p* = 0.081), but their peak velocity was significantly lower than in young adults (*p* = 0.015) and in children (*p* = 0.015).

The distance and the interaction factors did not show significant effects for all the velocity parameters (Table 4, rows 8–11).

Taken together, these results show that most of the changes in duration and velocity parameters occurred during the turning phases of the iTUG test, with increases in duration of time and reductions in velocity focused on the elderly group with respect to the other groups.

Furthermore, except for the obvious differences in time when duration was measured along 3 m or 7 m (total time, walking forward, and walking back), the only parameter sensitive to the distance was the duration of mid turning.

## 4. Discussion

The possibility to quantitatively explore the subcomponents of the iTUG test, by using a single wearable sensor, allowed us to observe specific kinematic changes associated with healthy aging and distance traveled; walking slowed down during the mid and final turning phases in the subjects over 70 years old, while walking speed increased during mid turning, for all the groups, throughout the longer path traveled (7 m vs. 3 m).

### 4.1. The Effects of Age Observed by the iTUG Test 

The similar responses observed in children, young adults, and senior adults indicate that the appropriate adaptations to face gross motor functions included in the TUG test are already developed in age period of 7–12 years and maintained up to 70 years old. 

This picture is coherent with the data from a number of papers reviewed by Iosa et al. [33] on the changes in upright gait stability throughout life. The stabilization of walking speed, and the acceleration along anterior-posterior and medial-lateral axes start at an age of 7–9 years, decay slowly throughout adult age, and worsen over 70 years old.

This observation is not surprising, because previous electrophysiological and biomechanical studies showed that individuals reach an adult pattern of gait and posture at around the age of 5–7 years [34]; the sensory-motor system is then sufficiently mature to allow postural adaptations with respect to extra personal space [35].

However, while a stabilization of mobility from middle childhood to adult age can be considered reasonable when common gross motor functions are evaluated by whole-body parametrization, we cannot exclude that movement strategies at the joint and/or muscle levels could be modulated during adolescent age in order to optimize movement costs, without significant changes in overall outcomes [36].

Thus, our results support the reliability of the iTUG as a test to assess the typical development within the range of middle childhood age (7–12 years), and they give value to the possibility of using this test to compare typically developed children with children with reduced mobility.

On the other hand, the elderly group showed a significant decrease in walking speed during mid turning and when turning before sitting down. This result supports the idea that quantification of single iTUG components is important to identify and characterize the specific physical ability associated with age.

The mid and final turning of the iTUG test are quite different tasks; the mid turning requires the ability to turn while walking, while the final turning consists of a few small steps followed by a pivot turn.

The caution showed by the elderly group in approaching mid turning of the iTUG test may depend on the kinematics and kinetics associated with change in direction while walking. Turning is a repeated and asymmetric task, requiring an accurate active control from the central nervous system because the progression of the center of mass is rapidly halted and redirected over the base of support. In fact, turning requires kinematics and force generation to rapidly reorient the head, trunk, and pelvis, with greater medial ground reaction impulses on the outside limb to lead the body center of mass upon the contralateral limb [37,38,39]. 

Enlarging the base of support along the medial-lateral axis is a postural adaptation adopted by elderly adults to guarantee appropriate postural reactions for greater safety in walking along a curved path [38,40,41]. On these bases, the slow down observed in elderly adults during mid turning may contribute to accomplish the medial-lateral expansion of the base of support, increasing the margins of stability while walking during turning.

The age-related changes observed during final turning should depend on different control processes with respect to mid turning. In fact, as mid turning can be considered a measure of the postural interaction between walking and turning [42], the small steps and the pivot turn, accomplished during final turning, are strongly associated with the stability required for an efficient and safe stand-to-sit phase. Therefore, the motor control of final turning should include important anticipatory components to compensate, in advance, for the postural destabilization intrinsic in the action of stand to sit. 

The slow down observed in elderly adults during final turning may compensate for a deterioration of anticipatory postural adjustments, as reported in other postural contexts [43,44]. In line with this hypothesis are the data reported by Weiss et al. [24], which found that 80% of elderly adults sampled performed final turning and sat down mainly as distinct tasks, increasing the time of execution with respect to the remaining 20% of the sample, who began to sit before they completed the final turn, overlapping the two tasks.

### 4.2. The Effects of Distance in Performing the iTUG Test

An interesting result that emerged in the current study is that the velocity of walking during mid turning increased when the iTUG test was performed along the 7 m distance. As there were no statistical differences over the groups or for the interaction factor, this result must be placed within the more general issue of the relationships between turning and the associated processes occurring during the previous linear walking. Many papers addressed this issue, finding several factors influencing an optimal walking while turning, for example, turning angle and velocity [45,46], height of obstacle to turn [47], and cognitive or manual secondary tasks [48]. To the best of our knowledge, data on the influence of different walking distances on turning behavior are not available. 

A possible explanation for the differences in speed during mid turning observed in our experiments could be that longer walking, more than 3 m, would give the time to better prepare for the turning task. For example, a greater walking space would increase the visual information concerning the feature of the turning point and elaborate a predictive behavior. This hypothesis is supported by a number of studies showing that the amount of visual sampling is an important factor when gait control requires representing specific trajectory changes in advance [49,50,51]. 

Notwithstanding the generic aspect of this result, it suggests that the importance of increasing the traveled distance during the TUG test is more than simply ensuring a sufficient set of gait parameters, as proposed by Zampieri et al. [22], but it is also useful to reveal functional mechanisms, such as those responsible for preparation and programming of actual movement.

In particular, specific gait disturbances, as occur in patients with cerebral palsy or muscle dystrophies, or with deficits in anticipatory adjustments, such as in Parkinson disease, could make some disturbances of motor programming overt during the execution of the 7 m walk using the iTUG test, but not during the 3 m walk. 

### 4.3. Benefits of Using Single Sensor to Perform the iTUG Test

In recent decades, the limitations of the TUG test in quantifying displacement and mobility were overcome thanks to motion analysis systems. Many of these systems require bulky and very expensive devices. Currently, the use of wearable sensors improved the parameterization by increasing the areas in which the TUG test can be used.

In this study, the instrumentation was reduced to a single sensor. To our knowledge, only one paper used a single sensor to evaluate the effects of aging by the TUG test [20]. However, in that work, children were not included, and the sampled subjects were distributed in two groups with an overlap between older and young adults.

We believe that the results of our study may contribute to the idea that the use of a minimum instrumentation, as a single sensor, may be adequate to extract sufficient data when the iTUG is used, as in the current work, to explore a more comprehensive age range or different levels of complexity. 

## 5. Conclusions

Similarities and differences in physical mobility from middle childhood to older adults and between two walking distances were exposed in this study, providing insights into the potential use of the instrumented TUG test. In fact, extending measurements from the entire total time to each subcomponent of the test allowed us to formulate functional significances in both the presence and the absence of differences. 

Children, as well as young and senior adults, exhibited similar values for total time and the parameters of each subcomponent, indicating that healthy individuals develop early the abilities associated with the tasks required by the TUG test. On the other hand, the changes in speed during the two turning phases shown by the elderly group, and during mid turning, comparing 3 m with 7 m distances, may be epiphenomena of a number of underlying mechanisms. However, the specific points where these variations occurred in the context of the TUG test suggest that elderly adults implement reactive postural adaptations, in the case of mid turning, and anticipatory postural adjustments, in the case of final turning. Similarly, preparatory adaptations may explain the increasing speed when the iTUG test was performed over 7 m.

Although these basic functional interpretations would require more accurate measurements and analyses, this study showed that the iTUG test can provide essential information for a more complete description of physical mobility across lifespan and when the test is performed at different walking distances. We believe that the large range of healthy people studied in this work and the use of a single wearable sensor make the current results useful in establishing rehabilitation programs, particularly for specific abnormalities associated with turning ability and walking length.

## Figures and Tables

**Table 1:** Anthropometric data.

Groups	Age Range (years)	Age (years)	Weight (kg)	Height (cm)
Children	7–12	9.27 ± 1.80	36.24 ± 10.08	139.68 ± 12.21
Young adults	31–40	34.15 ± 3.15	70.75 ± 11.66	167.63 ± 8.64
Senior adults	60–69	64.20 ± 2.98	74.09 ± 13.02	164.83 ± 7.39
Elderly persons	70–85	76.28 ± 4.06	71.56 ± 10.72	160.48 ± 10

Values are expressed as means ± standard deviation, except for age range.

**Table 2:** Original un-normalized temporal parameters.

Temporal Parameters	Total Time (s)	Sit to Stand (s)	Walking Forward (s)	Mid Turning (s)	Walking Back (s)	Final Turning (s)	Stand to Sit (s)
**3-m iTUG**							
Children	9.2 ± 0.8	1.2 ± 0.1	2.3 ± 0.4	1.6 ± 0.2	1.9 ± 0.3	1.2 ± 0.3	1.4 ± 0.3
Young adults	10.5 ± 1.1	1.4 ± 0.2	2.2 ± 0.4	2.1 ± 0.5	2.0 ± 0.6	1.6 ± 0.3	1.9 ± 0.4
Senior adults	10.5 ± 1.5	1.4 ± 0.2	2.3 ± 0.5	2.1 ± 0.3	2.1 ± 0.5	1.5 ± 0.3	1.8 ± 0.3
Elderly persons	11.4 ± 1.3	1.4 ± 0.2	2.5 ± 0.4	2.4 ± 0.4	2.2 ± 0.7	2.0 ± 0.5	1.9 ± 0.4
Correlation with weight and height (*r*/*p*)							
Un-normalized	**0.43/<0.001**	**0.44/<0.001**	**0.28/0.047**	**0.43/<0.001**	0.20/0.199	**0.43/<0.001**	**0.50/<0.001**
Normalized	0.007/0.997	0.011/0.995	0.032/0.962	0.028/0.970	0.036/0.951	0.039/0.943	0.011/0.995
**7-m iTUG**							
Children	15.9 ± 1.4	1.2 ± 0.1	5.5 ± 0.8	1.6 ± 0.3	5.4 ± 0.4	1.3 ± 0.3	1.3 ± 0.3
Young adults	17.0 ± 1.7	1.4 ± 0.2	5.4 ± 0.7	2.1 ± 0.4	5.3 ± 0.6	1.6 ± 0.3	1.8 ± 0.4
Senior adults	17.0 ± 2.3	1.4 ± 0.2	5.5 ± 1.1	2.0 ± 0.3	5.3 ± 0.9	1.6 ± 0.3	1.7 ± 0.2
Elderly persons	18.1 ± 2.0	1.4 ± 0.2	5.9 ± 0.9	2.2 ± 0.3	5.7 ± 0.9	1.9 ± 0.4	2.0 ± 0.5
Correlation with weight and height (*r*/*p*)							
Un-normalized	**0.32/0.019**	**0.46/<0.001**	0.23/0.122	**0.53/<0.001**	**0.30/0.031**	**0.40/0.001**	**0.50/<0.001**
Normalized	0.022/0.981	0.003/1	0.029/0.967	0.044/0.930	0.037/0.949	0.027/0.973	0.037/0.950

The temporal parameters are expressed in seconds (s) reporting means ± standard deviation. The correlations are validated by the coefficient of correlation (*r*) and the level of significance (*p).* Data reported in bold indicate statistically significant values.

**Table 3:** Original un-normalized velocity parameters.

Velocity Parameters	Average Mid Turning (°/s)	Peak Mid Turning (°/s)	Average Final Turning (°/s)	Peak Final Turning (°/s)
**3-m iTUG**				
Children	102.8 ± 14.2	205.6 ± 32.7	132.6 ± 26.2	255.7 ± 39.9
Young adults	84.3 ± 15.8	164.8 ± 23.6	104.7 ± 18.5	199.6 ± 30.98
Senior adults	84.7 ± 9.9	146.0 ± 25.1	105.0 ± 21.1	189.3 ± 36.2
Elderly persons	72.5 ± 10.3	134.4 ± 20.3	84.5 ± 19.9	168.4 ± 31.9
Multiple regression with weight and height (*r*/*p*)				
Un-normalized	**0.52/<0.001**	**0.59/<0.001**	**0.54/<0.001**	**0.60/<0.001**
Normalized	0.038/0.945	0.062/0.863	0.050/0.908	0.029/0.969
**7-m iTUG**				
Children	102.2 ± 15.83	207.8 ± 37.83	134.5 ± 26.8	268.29 ± 40.5
Young adults	84.9 ± 14.4	164.6 ± 19.5	105.6 ± 16.5	202.5 ± 36.81
Senior adults	84.2 ± 9.9	152.9 ± 23.7	103.6 ± 18.6	191.07 ± 35.0
Elderly persons	76.9 ± 10.0	138.5 ± 18.9	85.8 ± 14.5	167.6 ± 24.1
Multiple regression with weight and height (*r*/*p*)				
Un-normalized	**0.53/<0.001**	**0.63/<0.001**	**0.53/<0.001**	**0.65/<0.001**
Normalized	0.04/0.941	0.075/0.804	0.041/0.938	0.053/0.896

The velocity parameters are expressed in degrees per seconds (°/s) reporting means ± standard deviation. The correlations are validated by the coefficient of correlation (*r*) and the level of significance (*p).* Data reported in bold indicate statistically significant values.

**Table 4:** Summary of the two-way analysis of variance; df—degrees of freedom.

Parameters	Groups df: 3.76		Distances df: 1.76		Groups X Distances df: 3.76	
	*F*	*p*	*F*	*p*	*F*	*p*
1. Total time	2.461	0.069	3617.228	**<0.001**	3.648	**0.016**
2. Sit to stand duration	0.155	0.926	0.423	0.518	0.115	0.951
3. Walking forward duration	1.821	0.150	1982.924	**<0.001**	0.178	0.911
4. Walking back duration	0.477	0.699	2778.295	**<0.001**	1.768	0.161
5. Stand to sit duration	0.811	0.492	2.700	0.104	0.428	0.734
6. Mid turning duration	2.409	**0.040**	5.322	**0.024**	0.630	0.598
7. Final turning duration	5.690	**0.001**	0.653	0.422	1.109	0.351
8. Average mid turning velocity	4.081	**0.010**	2.719	0.103	1.117	0.347
9. Average final turning velocity	5.288	**0.002**	0.057	0.940	0.053	0.984
10. Peak mid turning velocity	4.741	**0.004**	1.910	0.171	0.535	0.659
11. Peak final turning velocity	4.503	**0.006**	0.265	0.264	1.069	0.367

The F-statistic and the *p*-values were not adjusted applying Greenhouse-Geisser correction, since no violation of sphericity was detected (Mauchly’s test). Data reported in bold indicate statistically significant values. Abbreviation: df, degrees of freedom.
